# Supplementary material for: Genetic Analysis of Candida albicans Filamentation by the Iron Chelator BPS Reveals a Role for a Conserved Kinase—WD40 Protein Pair
Source: J Fungi (Basel). 2024 Jan 22;10(1):83. doi: 10.3390/jof10010083 (PMC10820326; doi:10.3390/jof10010083)
Supplement: Supplementary file 1 [file jof-10-00083-s001.zip › Supplementary Figures for Pinsky et al.pdf]

Supplementary Figures for Pinsky et al.

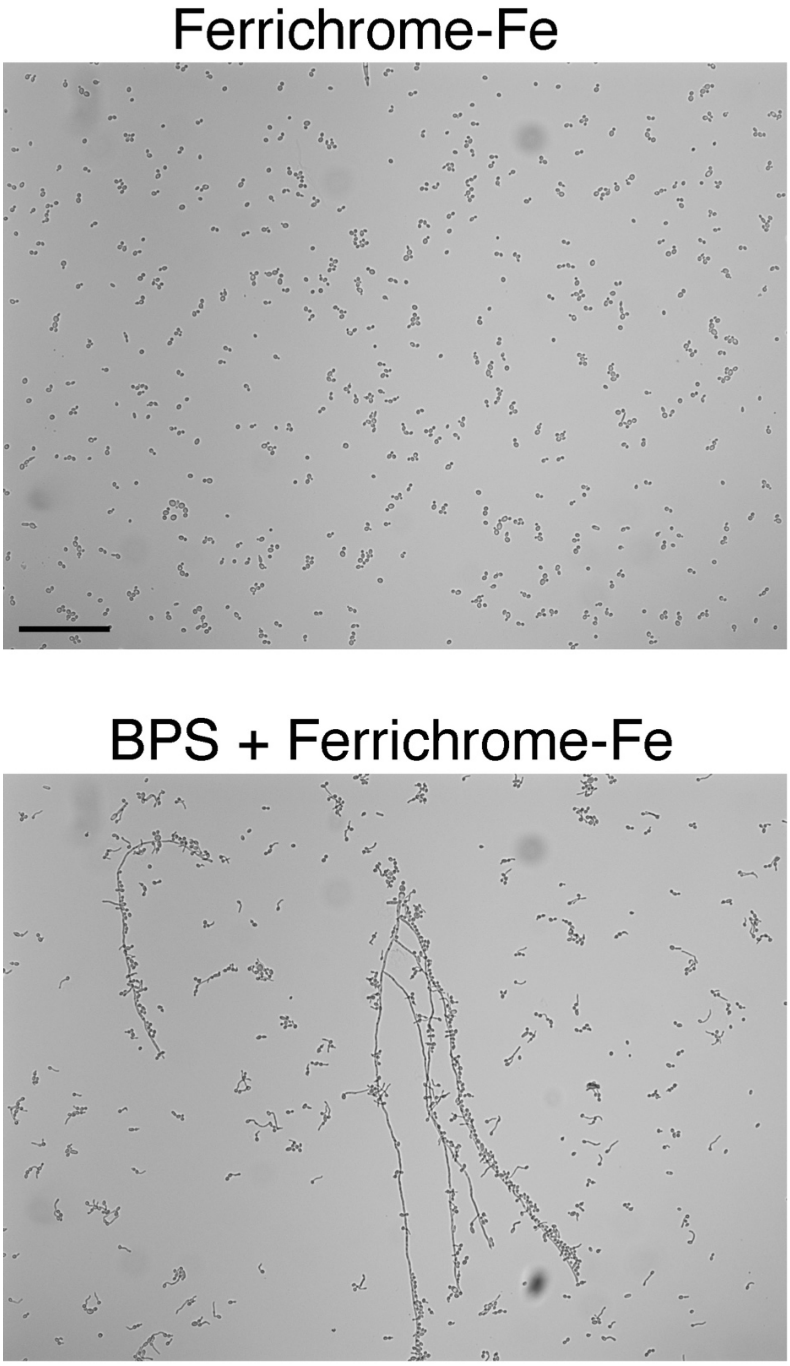

Figure S1. Partial rescue of BPS growth inhibition by iron-ferrichrome induces filamentous growth. Strain KC1175 was diluted to OD600 = 0.002 in a 0.1 ml well-aerated culture and grown 1 day in YPD + 100 µM ferrichrome pre-loaded with 80 µM $FeCl_3$ (top) or 2 days in YPD + 2 mM BPS + 100 µM ferrichrome pre-loaded with 80 µM $FeCl_3$ (bottom). The cells were visualized with a 10x objective. Scale bar = 100 µm.

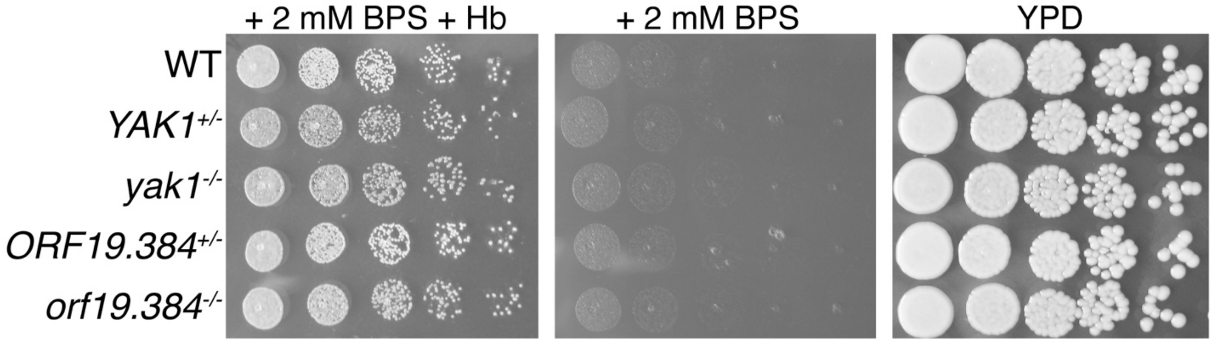

Figure S2. The *yak1* and *orf19.384* mutants are not defective in growth on BPS + hemoglobin. The indicated strains, wild-type (KC1175), *YAK1*$^{+/-}$ (KC1336), *yak1*$^{-/-}$ (KC1338), *ORF19.384*$^{+/-}$ (KC1340), and *orf19.384*$^{-/-}$ (KC1363), were sequentially diluted and inoculated as drops on YPD plates without or with 2 mM BPS and 1 μM hemoglobin. The plates were photographed after 3 days of incubation at 30°C.

Figure S3. (next page) Effect of deletion of *YAK1* or *ORF19.384* on expression and localization of their partner protein. Wild-type (KC1669) and *orf19.384*$^{-/-}$ (KC1672) cells expressing *YAK1* fused to GFP, and wild-type (KC1670) and *yak1*$^{-/-}$ (KC1671) cells expressing *ORF19.384* fused to GFP were grown to log phase in SC-URA medium, incubated 5 min with Hoechst 33342 for nuclear staining, and visualized by DIC and epifluorescence microscopy with a 100X objective. C= untagged control strain KC1175. Scale bar = 5 μM.

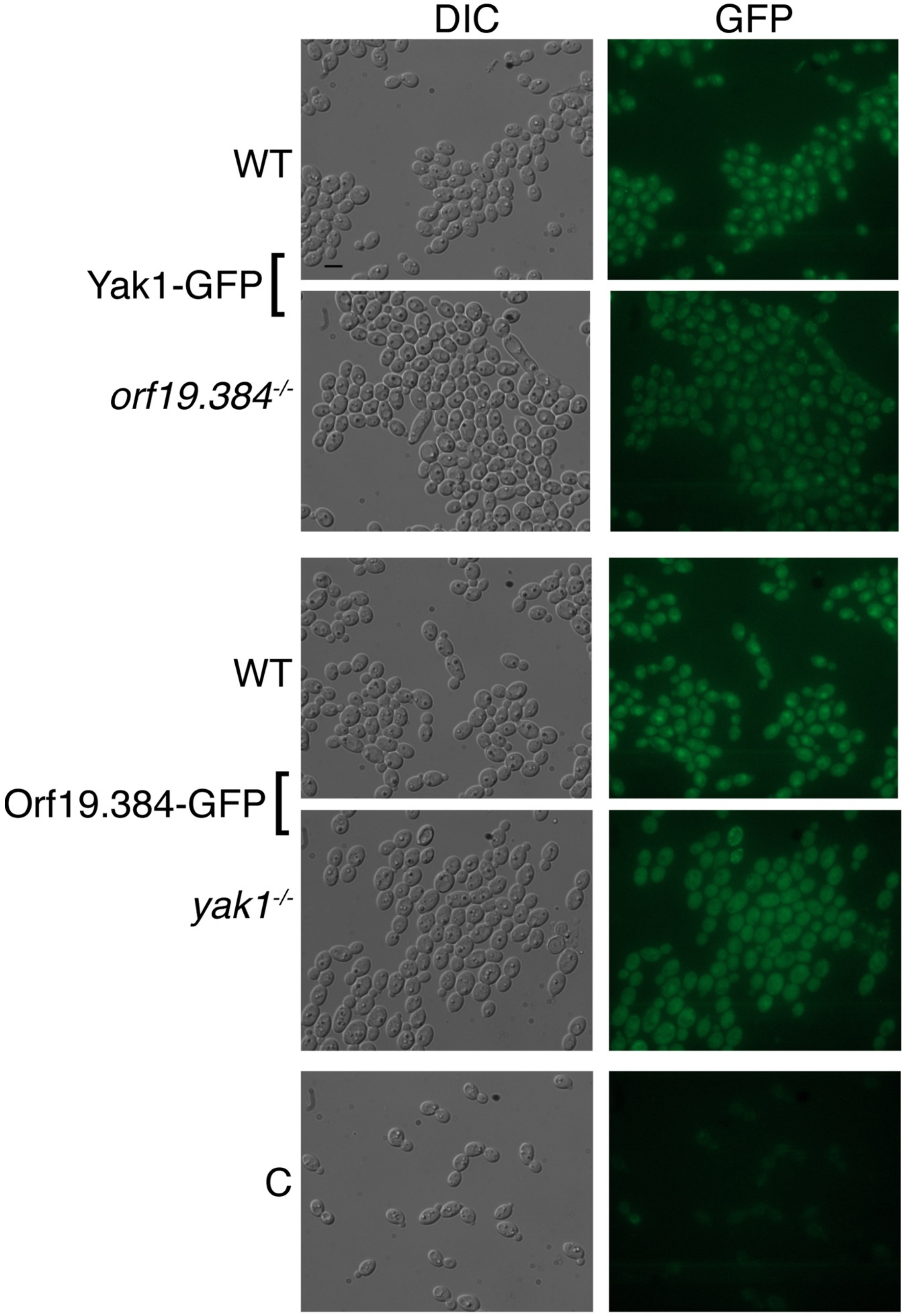

DIC
GFP
WT
Yak1-GFP
orf19.384$^{-/-}$
WT
Orf19.384-GFP
yak1$^{-/-}$
C
