# Supplementary material for: Genetic Analysis of Candida albicans Filamentation by the Iron Chelator BPS Reveals a Role for a Conserved Kinase—WD40 Protein Pair
Source: J Fungi (Basel). 2024 Jan 22;10(1):83. doi: 10.3390/jof10010083 (PMC10820326; doi:10.3390/jof10010083)
Supplement: Supplementary file 1 [file jof-10-00083-s001.zip › Table S1 - Primers.pdf]

Table S1: List of primers

| # | Name | Sequence | Direction |
|---|---|---|---|
| **1** | **F1-Transposon-FPNI** | CCGTTCGTTTTCGTTACCGGTATATC | Forward |
| **2** | **F2-Transposon-FPNI** | CCGTCCCGCAAGTTAAATATG | Forward |
| **3** | **F3-Transposon-FPNI** | GTATTTTACCGACCGTTACCGACC | Forward |
| **4** | **R1-FPNI** | CGCAGGTGACATAGATGC | Reverse |
| **5** | **R2-FPNI** | GACATAGATGCTTAGCGCTGAGG | Reverse |
| **6** | **R3-FPNI** | CGCAGGTGACATAGATGCTTAGCGCTGAG GNNNNNNNNN | Reverse |
| **7** | **PstI-YAK1(+610)** | gcctgcagTTGGGTAGTAATGACCG | Forward |
| **8** | **YAK1(+2427)-SalI** | atgtcgacTAAATCAGTGGTATTAACATCAATA AT | Reverse |
| **9** | **SacI-5'YAK1KO(-400)** | acgagctcACACCAGACAGACTACA | Forward |
| **10** | **5'YAK1KO(+80)-SpeI** | gcactagtTGCCAATTTCCTCCTAT | Reverse |
| **11** | **PstI-3'YAK1KO(+2370)** | acctgcagAACACCTCAAGAAGCTT | Forward |
| **12** | **3'YAK1KO(+3047)-XhoI** | gcctcgagCGTCCAAATGTAGATTGTAA | Reverse |
| **13** | **PstI-CaORF19.384(+302)** | gcctgcagTTCCTCCAACACCATTAG | Forward |
| **14** | **CaORF19.384(+1674)-SalI** | atgtcgacTATCAGAACTGCTTGGAAAC | Reverse |
| **15** | **SacI-5'ORF19.384KO(-273)** | acgagctcTTTGTGCCCGTTGGTAA | Forward |
| **16** | **5'ORF19.384KO(+80)-SpeI** | gcactagtCCATGAAATGCTGGGTT | Reverse |
| **17** | **PstI-3'ORF19.384KO(+1607)** | acctgcagGTTGGAGACAGAACCAA | Forward |
| **18** | **3'19.384KO(+2096)-XhoI** | gcctcgagGCTATCGTCAAGATGGT | Reverse |
| **19** | **F-Test-YAK1** | GGAATGGAATGATAACG | Forward |
| **20** | **5'hisG** | cggaaccctgtgatggag | Reverse |
| **21** | **F-Test-ORF19.384** | TGCTTACCCATTACCAT | Forward |
| **22** | **3'hisG** | ggctttctcatgcgttcatg | Reverse |
| **23** | **5pSFL1-13xMyc** | AATAGTGATGACAATGGGAACGATCATAAA AAGAGAAAATTAGAACGG ATC CCC GGG TTA ATT AA | Forward |
| **24** | **3pSFL1-13xMyc** | TTTGTAAAACATAGTGTCATTTTTTCAAGTT TTAATTAAATTTACtctgatatcatcgatgaattcgag | Reverse |
