# Supplementary material for: Genetic Analysis of Candida albicans Filamentation by the Iron Chelator BPS Reveals a Role for a Conserved Kinase—WD40 Protein Pair
Source: J Fungi (Basel). 2024 Jan 22;10(1):83. doi: 10.3390/jof10010083 (PMC10820326; doi:10.3390/jof10010083)
Supplement: Supplementary file 1 [file jof-10-00083-s001.zip › Table S2 - quantitation of Figure 2.pdf]

Table S2 – quantitation of Fig. 2

Strain	Field 1	Field 2	Field 3	Average
KC2 = CAI1 ura+	66%	90%	95%	85%
KC148 = *cph1*$^{-/-}$	18%	25%	15%	19%
KC149 = *efg1*$^{-/-}$	0%	0%	0%	0%
KC445 = *ume6*$^{-/-}$	0%	0%	0%	0%
KC532 = *hgc1*$^{-/-}$	0%	0%	0%	0%
Homann WT	38%	53%	64%	52%
Homann *ndt80*$^{-/-}$	15%	17%	22%	18%
Homann *sfl2*$^{-/-}$	18%	19%	23%	20%
Homann *rob1*$^{-/-}$	0%	0%	0%	0%
Homann *tec1*$^{-/-}$	33%	31%	25%	30%
Homann *cph2*$^{-/-}$	55%	50%	66%	57%
Homann *hap43*$^{-/-}$	56%	37%	51%	48%

Percentage hyphae in the cultures shown in Figure 2 was obtained by photographing three different fields with the 10X objective and counting the percentage of hyphae in each field. Of note, the less-filamenting mutants typically also had shorter hyphae, as can be seen in Figure 2.
